# A prospective longitudinal population-based cohort study of non-communicable diseases (NCDs) in Ghana, India, and Nepal (The GIN NCDs Study): A study protocol

**DOI:** 10.1371/journal.pone.0362114

**Published:** 2026-11-12

**Authors:** Padam Simkhada, Bibha Simkhada, Rajeeb Kumar Sah, John Stephenson, Edwin van Teijlingen, Ak Narayan Poudel, Zoe Darwin, Devendra Raj Singh, Chia-Yuan Lin, Philip Brown, Vijay S GC, Shital Telrandhe, Joshua Okyere, Alfred Edwin Yawson, Eric Nana Yaw Nyarko, Elom Hillary Otchi, Tribikram Budhathoki, Mahalaqua Nazli Khatib, Abhay Gaidhane, Zahir Quazi, Bikash Koirala, Precious Adade Duodu, Ram Chandra Silwal, Arjun Aryal, Rajan Paudel, Sanjay Poudel, Pramod Regmi, Sujan Simkhada, Abhishek Silwal, Priyamvada Paudyal, Lalita Kumari Sah, Dale Grant, Paul Bissell, Kaung Suu Lwin

**Affiliations:** 1 University of Chester, United Kingdom; 2 School of Human and Health Sciences, University of Huddersfield, United Kingdom; 3 Bournemouth University, United Kingdom; 4 Centre for Health Economics, University of York, York, United Kingdom; 5 Adani Institute of Higher Education & Research, India; 6 University of Ghana, Ghana; 7 University of Ghana, School of Public Health, Department of Health Policy Planning & Management, Korle-Bu Teaching Hospital, Accra, Ghana; 8 Queen Mary University of London, United Kingdom; 9 Green Tara Nepal, Nepal; 10 Tribhuvan University, Nepal; 11 School of Medicine, Keele University, United Kingdom; 12 Faculty of Health and Social Care, University of Bradford, United Kingdom; Freelance Consultant, Myanmar, MYANMAR

## Abstract

**Background:**

Non-communicable diseases (NCDs) are the leading cause of death globally, disproportionately affecting low-and-middle-income countries (LMICs). In Ghana, India, and Nepal, the NCD burden is rising due to urbanisation, demographic shifts, and lifestyle changes. Existing national datasets are largely cross-sectional, limiting insights into disease progression and intergenerational health patterns. Longitudinal evidence is urgently needed to inform prevention and care strategies.

**Methods:**

All residents aged ≥18 years residing in the study areas will be invited to enrol at baseline, with plans to include younger age groups in future waves. Data will be collected at baseline, Year 2, and Year 4 using harmonised tools adapted from the World Health Organization’s STEPwise approach to Surveillance (WHO STEPS) instruments. Domains include socio-demographics, climate related exposures and impacts, lifestyle factors, health-seeking behaviours, mental and physical health, and physical and anthropometric measurements. Primary outcomes include hypertension, diabetes, chronic respiratory diseases, mental health disorders, dementia, and multimorbidity. The cohort is powered to estimate the prevalence of raised blood pressure with ±3% precision, assuming a design effect of 1.5, 10–15% non-response, and 10% annual attrition. Required baseline sample sizes are calculated as: 2,211 (India), 2,126 (Nepal), and 2,262 (Ghana). To achieve precision within rural, peri-urban, and urban strata, these totals will triple to 6,628 (India), 6,378 (Nepal), and 6,786 (Ghana). Sampling will follow a two-stage cluster design with household-level enrolment. Analyses will use multivariable mixed-effects models guided by a directed acyclic graph (DAG) framework.

**Discussion:**

This study will generate longitudinal evidence on NCD risk factors, multimorbidity, and mental health in LMICs, addressing critical data gaps. Its household-based design enables exploration of shared exposures and intergenerational influences, supporting life-course analyses. Findings will inform targeted interventions, health system planning, and policy frameworks aligned with the WHO Global Monitoring Framework and Sustainable Development Goal (SDG) Target 3.4.

## Background and rationale

Non-communicable diseases (NCDs), including cardiovascular disease (CVD), diabetes, chronic respiratory diseases and cancers are now the leading causes of death globally, responsible for approximately 74% of all deaths, with over three-quarters of all NCD deaths occurring in low-and-middle-income countries (LMICs) [1]. In Ghana, India, and Nepal, rapid urbanisation, demographic shifts, and changes in dietary and lifestyle behaviours have accelerated the NCD epidemic, straining health systems and exacerbating health inequities.

In Ghana, home to about 35.1 million people, the 2023 WHO STEPS survey revealed that 45% of all deaths are attributable to NCDs, with cardiovascular disease (CVD) as a major contributor [2]. Hypertension, a key metabolic risk factor for CVD, is widespread, yet more than half of hypertensive individuals remain unaware of their condition [2]. Other related risks include physical inactivity (14.6% of women and 5.5% of men), high salt intake (22.8% among young adults), and overweight or obesity (49.8% of women and 19.7% of men) [2]. These patterns reinforce evidence of a growing double burden of disease where populations face both NCDs and communicable diseases, particularly in urban populations [3,4]. Meanwhile, India, with a population of approximately 1.46 billion, sees NCDs accounting for more than 63% of all deaths, with CVD (28%), chronic respiratory disease (11%), and diabetes (3%) being the leading contributors [5]. Behavioural risk factors are prevalent across the country, including tobacco use, unhealthy diets, physical inactivity, and alcohol consumption [6]. According to the National Noncommunicable Disease Monitoring Survey (NNMS), 32.8% of men and 1.6% of women use tobacco, 18.8% of men and 1.3% of women consume alcohol, and 98.4% of adults consume fewer than five servings of fruits and vegetables per day [6]. Additionally, 41.3% of adults are physically inactive, and 26.1% are overweight or obese [6]. Metabolic risk factors are also widespread, with 28.5% of adults having raised blood pressure and 9.3% having raised fasting blood glucose [6]. Clustering of multiple risk factors is common, particularly in urban and aging populations, with 50.1% of adults in some regions exhibiting three or more NCD-related risk factors [6]. Although India has generated large-scale datasets such as NNMS, the National Family Health Survey (NFHS-5) [7] and the Longitudinal Ageing Study in India (LASI) [8], these are either cross-sectional or focused on specific age groups. In Nepal, which has a population of around 29.6 million, NCDs account for approximately 66% of all deaths [9]. The 2019 STEPS survey revealed that 24.5% of adults had elevated blood pressure, 5.8% had raised blood glucose, 24.3% were overweight or obese, and nearly all (97%) consumed insufficient fruits and vegetables [10]. One in four adults had three or more risk factors, with many remaining undiagnosed and untreated.

Most existing national health datasets in Ghana, India, and Nepal are cross-sectional, limiting their ability to examine disease progression, changes in risk exposure, and intergenerational health patterns over time. Longitudinal evidence is needed to better understand how NCDs develop, cluster, and interact across the life course within diverse sociocultural and economic contexts. Ghana, India, and Nepal were selected to capture variation in NCD epidemiology, demographic transition, urbanisation, socioeconomic development, and health-system capacity across LMIC settings. Although the countries differ in demographic characteristics, health-system contexts, and stages of epidemiological transition, they share a rapidly increasing NCD burden driven by common behavioural and metabolic risk factors, including unhealthy diets, physical inactivity, tobacco use, obesity, hypertension, and diabetes [2,6,10]. Studying these settings using harmonised methods will enable comparison of both shared and context-specific determinants of NCDs, helping to identify factors that are consistent across populations as well as those shaped by local social, environmental, and health-system contexts. Although not intended to be representative of all LMICs, the inclusion of countries from South Asia and sub-Saharan Africa provides an opportunity to generate transferable evidence on NCD prevention and control across diverse settings. The countries were also selected based on practical considerations essential for establishing a sustainable longitudinal cohort, including the availability of strong local research partnerships, existing field infrastructure, experienced research teams, and institutional commitment to support long-term participant follow-up and high-quality data collection.

Beyond behavioural and metabolic risk factors, Ghana, India, and Nepal are also affected by broader environmental and social determinants of NCDs. Air pollution has emerged as an important contributor to cardiovascular and respiratory disease across all three countries. In Nepal, exposure to ambient and household air pollution is associated with substantial burdens of ischaemic heart disease, stroke, chronic respiratory disease, and lung cancer, while air pollution in Ghana contributes to significant cardiovascular and respiratory morbidity and mortality [11,12]. In India, air pollution is increasingly recognised as a major contributor to cardiovascular and respiratory diseases and acts alongside rapid urbanisation and lifestyle changes to accelerate the NCD burden [13]. Socioeconomic inequalities also play an important role in shaping NCD risk and outcomes across the three countries. Evidence from India, Ghana, and Nepal demonstrates disparities in exposure to risk factors, healthcare access, diagnosis, treatment, and health outcomes according to wealth, education, place of residence, and other social determinants [14–16]. These shared challenges, together with differences in environmental exposures, patterns of urbanisation, and health-system contexts, provide a valuable opportunity to investigate both common and context-specific determinants of NCDs using harmonised longitudinal data.

This study aims to establish longitudinal cohorts in defined catchment areas in each country, beginning with adults aged 18 and above, and expanding to include all age groups over time. By enrolling entire households and collecting repeated, harmonised data, the study will enable analysis of behavioural, social, and environmental determinants of NCDs—capturing not only individual trajectories but also family-level influences. Findings will inform targeted interventions and policies to strengthen prevention and care efforts, supporting countries’ commitments to Universal Health Coverage (UHC) and the Sustainable Development Goals (SDGs), particularly Target 3.4 which aims to reduce premature mortality from NCDs by one third by 2030 [17].

## Aim

To establish longitudinal population-based cohorts in selected communities in Ghana, India, and Nepal to examine the development and progression of NCDs and their associated risk factors

## Objectives

1. To measure the period prevalence of major NCDs (hypertension, diabetes, chronic respiratory diseases, dementia and mental health disorders) and associated risk factors;
2. To track changes in behavioural, environmental and social determinants of NCDs over time through repeated data collection;
3. To identify vulnerable population subgroups at higher risk of NCDs and their risk factors, disaggregated by age, sex, geography, and socioeconomic status;
4. To generate evidence to inform health policy and community-level interventions aimed at NCD prevention, early detection, and equitable access to care.

## Methods

### Study design

This is longitudinal, population-based cohort study, in selected catchment areas in Ghana, India, and Nepal, that will examine the development, progression, and clustering of NCDs and their associated risk factors over time.

The initial study period will span four years, supported by current funding, and plans to extend the cohort beyond this period to capture long-term NCD trajectories. Data from the participants will be collected at multiple time points:

- At Year 0 (Baseline), a comprehensive assessment will be conducted, including demographic, behavioural, physical, and anthropometric measurements.
- In Year 1 and Year 3, brief follow-up visits will be conducted to update vital status, hospitalizations, medication use, and contact information.
- At Year 2 (Midline) and Year 4 (Endline of the funded phase), full follow-up assessments will be repeated to evaluate changes in health status, risk factors, and incidence of NCDs.

We plan to seek external funding to extend the study beyond Year 4 and will continue to follow the participants through annual brief check-ins and biennial full assessments (in Years 6, 8, etc.). The study team will explore the use of passive data collection methods, such as health record linkage or mobile health tools, to enhance follow-up efficiency in the extended phase. It will also assess the feasibility of such linkage, including the availability of data infrastructure, unique identifiers, and governance frameworks, recognising that this may represent a longer-term development in some settings.

### Study population and setting

The study will take place in selected rural, peri-urban, and urban catchment areas across Ghana, India, and Nepal based on the degree of urbanisation defined by Organisation for Economic Co-operation and Development (OECD) [18]. Study sites have been chosen to reflect a range of geographic, socioeconomic, and health system contexts, enabling the study to capture variation in exposure to risk factors and access to care.

In Ghana, the study will be conducted in Ga East Municipality in the Greater Accra region that represent a range of peri-urban and rapidly developing settings with varying socioeconomic profiles and healthcare access. In India, the study will be conducted in Wardha and Seloo blocks of Wardha District, Maharashtra, which encompass urban, peri-urban, and rural communities with varying socioeconomic characteristics and access to healthcare services. In Nepal, the study will be conducted in Nagarjun Municipality, Kathmandu District, Bagmati Province, which includes communities with diverse socioeconomic characteristics and varying levels of urbanisation and service availability. Together, these sites provide an opportunity to examine how social, environmental, and health-system factors shape NCD risk, progression, multimorbidity, and access to care across different population groups. Initially, all residents in study areas aged 18 years and above will be eligible to participate, regardless of their NCD status, with plans to gradually include individuals of all ages. Individuals who are terminally ill, bedridden, or physically or mentally unable to provide informed consent or respond to survey questions will be excluded from data collection. For analysis and reporting purposes, participants will be grouped into the following age categories: 18–29, 30–44, 45–59, and ≥60 years. These categories reflect key stages of adulthood and will facilitate the assessment of age-related differences in NCD risk factors, disease prevalence, multimorbidity, and healthcare utilisation. Additional age categorizations may be used for specific analyses where appropriate.

Through household-level enrolment and repeated, harmonised data collection, the study will produce longitudinal insights into how behavioural, social, and environmental factors shape NCD risk and outcomes across different life stages and generations. Participation will be entirely voluntary and based on informed consent.

### Sampling and recruitment

A community census will be undertaken in each catchment to enumerate all households, using digital tools to support mapping, data entry, and quality assurance. This census will serve as a comprehensive sampling frame from which eligible individuals will be invited to participate. In the initial phase, recruitment will focus on residents aged 18 years and above, with plans to expand to all age groups if the study is extended beyond Year 4 through additional funding. Recruitment will be supported by trained local health workers and community leaders, coordinated through established partnerships with local research collaborators. Recruitment will be facilitated through door-to-door visits and community mobilization efforts, with informed consent obtained prior to participation. Data collection will include structured interviews using a standardized questionnaire, physical measurements, and random blood glucose measurements, conducted by trained field researchers following standardized protocols. Quality control procedures such as supervisor checks, real-time data monitoring, and periodic refresher training will ensure data integrity.

While the study is conducted within defined catchment populations in Ghana, India, and Nepal, the selected sites were chosen to capture a broad range of demographic, socioeconomic, environmental, and health-system contexts. The study aims to generate robust longitudinal evidence on NCD epidemiology, risk factors, and disease trajectories across diverse populations and settings. This diversity is a key strength of the study and enhances the external validity and generalisability of findings to populations with similar characteristics across LMIC contexts. Detailed contextual and epidemiological data, including the baseline prevalence and distribution of NCDs and associated risk factors, will support assessment of the applicability of findings across different settings. Analytical approaches such as weighting and statistical adjustment will also be applied, where supported by available population data, to account for differences between study populations and wider target populations. Findings will therefore be interpreted within their local contexts while providing insights that may inform NCD policy and programme development in comparable populations and health-system settings.

### Primary outcomes for sample size estimation

The cohort is powered to estimate the prevalence of hypertension (elevated blood pressure, BP) and raised random blood glucose at baseline, as these indicators are central to the global NCD monitoring framework [19]. Raised BP has been selected as the primary outcome for sample size determination because it is the most prevalent modifiable NCD risk factor in the target populations, ensuring adequate precision for other outcomes such as raised blood glucose [2,6,9,10].

Based on WHO STEPS surveys and published meta-analyses, the estimated prevalence of raised BP among those ≥18 is approximately 28.5% in India, 24.5% in Nepal, and 27% in Ghana [2,6,9,10].

### Sample size calculation

Sample size calculations assume a 95% confidence level and ±3% absolute precision for estimating the national prevalence of raised blood pressure. A design effect of 1.5 was applied to account for clustering, based on an average cluster size of 35 and an intraclass correlation coefficient (ICC) of 0.014. We further accounted for a baseline non-response rate of 10–15% and an annual attrition rate of 10% over four years, corresponding to an anticipated retention of approximately 66%.

### Sample size formula for proportion in cluster sampling


$$\begin{array}{c} n=\frac{Z^{\text{2}}.p.(\text{1}-p).DE}{d^{\text{2}}} \end{array}$$

Where:

- *n* = required sample size (before adjusting for non-response and attrition)
- *Z* = Z-score corresponding to the desired confidence level (i.e., 1.96 for 95%)
- *p* = estimated prevalence, as a proportion, i.e., 0.285 (28.5%) for India, 0.245 (24.5%) for Nepal, and 0.27 (27%) in Ghana
- *d* = desired absolute precision (i.e., 0.03 for ±3%)
- DE = design effect to account for cluster sampling

$$\begin{array}{c} n(adjusted)=\frac{n}{r.\alpha } \end{array}$$

- *r* = expected response rate (i.e., 90% → 0.90)
- *α* = expected retention over 4 years (i.e., 66% → 0.66)

Under these assumptions, with separate rural, peri-urban, and urban strata, the sample size per stratum will be calculated to maintain ±5% precision for raised BP prevalence, assuming similar prevalence across strata. This would require:

- India: 6,628 participants (2,209 per stratum)
- Nepal: 6,378 participants (2,126 per stratum)
- Ghana: 6,786 participants (2,262 per stratum)

These estimates ensure adequate power for stratified analyses and subgroup comparisons [20].

### Sampling strategy

A two-stage cluster sampling design will be employed:

1. First stage: Selection of clusters (communities) using probability proportional to size (PPS) sampling.
2. Second stage: Within each cluster, all eligible residents in sampled households will be invited to participate.

The average cluster size is expected to be 30–40 adults, subject to confirmation during pilot household listing. This approach balances statistical efficiency with operational feasibility [21].

### Data collection tools

Age-specific, structured questionnaires will be administered, adapted from established global survey instruments, including WHO STEPS, the Demographic and Health Surveys (DHS), LASI, and the Global School-based Student Health Survey (GSHS). The questionnaires will cover key domains including socio-demographic characteristics (including housing and household energy use), climate-related exposures and impacts, lifestyle and behavioural risk factors, psychosocial wellbeing and mental health, health-seeking behaviours and access to services, chronic disease symptoms and status, and functional status and quality of life. The questionnaires’ development and data interpretation will be informed by multidisciplinary subject matter experts with experience in cardiovascular disease, diabetes, mental health, epidemiology, public health, and health systems research.

Data collection will be conducted by trained field researchers recruited and supervised by local study teams and partner institutions in each country. Field researchers will be responsible for administering questionnaires, obtaining informed consent, and conducting physical measurements according to standardised study protocols. All field researchers will undergo comprehensive training and regular supervision to ensure consistency and data quality across study sites. Field researchers will be remunerated in accordance with local institutional policies and project budgets.

### Health outcomes and measurements

Depending on ethical and logistical feasibility, the study will assess both self-reported and objectively measured health outcomes related to NCDs and associated risk factors. Cardiovascular disease outcomes will include hypertension, assessed through measured blood pressure and/or self-reported diagnosis by a health professional, as well as self-reported history of stroke and heart disease. Blood pressure will be measured using validated automated digital blood pressure monitors following a standardised protocol across study sites, with regular equipment calibration and quality assurance procedures. Metabolic conditions will include diabetes mellitus, assessed through random blood glucose measurements and/or self-reported diagnosis, and overweight and obesity, assessed using body mass index (BMI). Blood glucose measurements will be conducted using standardised glucometers and protocols across study sites, with field researchers trained in infection prevention, biosafety procedures, and safe disposal of lancets and other sharps in accordance with local regulations. Chronic respiratory conditions will include self-reported history of asthma and chronic obstructive pulmonary disease (COPD). Where feasible, lung function will also be assessed using standardised peak expiratory flow measurements conducted according to harmonised protocols across study sites. Any differences in the availability of objective measurements will be documented and considered in the interpretation of findings.

Mental health outcomes will be assessed using the WHO STEPS depression and suicide modules [22], including measures of depressive symptoms and suicidal ideation or behaviour. These measures are intended to identify common mental health symptoms and population-level patterns rather than provide comprehensive diagnostic assessment of mental health disorders. The study will also examine multimorbidity, defined as the presence of two or more chronic conditions in the same individual. Cognitive impairment and dementia risk will be assessed using the 6-Item Cognitive Impairment Test (6CIT), a brief cognitive screening instrument with demonstrated accuracy for detecting cognitive impairment and dementia across clinical and community settings [23].

To support these assessments, trained field research teams will collect a range of physical and anthropometric measurements using portable equipment and standardised protocols. These measurements will include height and weight to calculate BMI, waist and hip circumference, blood pressure, heart rate, blood glucose measured through a finger-prick test where approved and feasible, peak expiratory flow, and cognitive function using the 6CIT. All measurements will follow standardised operating procedures, with appropriate training, supervision, and quality assurance processes to ensure consistency across study sites and countries.

### Data collection timeline

Participants will undergo a comprehensive assessment at baseline (Year 0), followed by brief interim follow-up visits in Years 1 and 3 to update vital status, hospitalisations, medication use, and contact information, and comprehensive assessments at Years 2 and 4.

Each round will include re-administration of questionnaires and physical measurements, with adjustments to capture time-varying exposures and outcomes. To minimise missing data, the study will implement proactive strategies including community engagement, flexible follow-up methods (e.g., home visits, telephone interviews), and a participant tracking system. Data quality will be ensured through standardised training for field teams, the use of harmonised instruments adapted from the WHO STEPS framework, and real-time electronic data checks during data collection.

The study is being implemented at different stages across the participating countries. In India and Ghana, the study is currently in the preparatory phase. Household listing and pilot testing are scheduled to commence on 20/08/2026. Participant recruitment in these two countries is expected to begin on 01/09/2026 and be completed by 30/11/2026. Data collection will run concurrently with recruitment and is anticipated to be completed by 28/02/2027.

In Nepal, preparatory activities have been completed, and participant recruitment and data collection commenced on 01/04/2026. Recruitment and data collection are currently ongoing and are expected to be completed by 28/02/2027.

Following completion of data collection, data cleaning and analysis will begin immediately. Preliminary study results are expected to be available by 30/06/2027. At the time of manuscript submission, no study results have been generated in any of the participating countries.

### Ethical considerations

Ethical approval for the study has been obtained from the relevant institutional review boards in Ghana, India, Nepal, and the coordinating institution, the University of Huddersfield, United Kingdom (Reference: SREIC/2025/067). All participants will provide informed consent prior to enrolment. Written consent will be obtained whenever possible; in cases where literacy is a barrier, verbal consent will be documented using a standardized form and witnessed by a trained field researcher. Participants will not receive financial incentives for participation. As a benefit of participation, individuals will receive feedback on selected health assessments, including anthropometric measurements, blood pressure and blood glucose measurements, and will be referred to appropriate healthcare services when clinically significant findings are identified. These procedures are intended to promote ethical conduct and participant welfare while minimising undue influence on participation. All study procedures will adhere to ethical principles and relevant national research governance guidelines. The study design has been informed by local expertise in each country through consultations with health professionals, community representatives, and research partners (S1 file). These discussions ensured that household survey methods, recruitment strategies, and the burden of participation are culturally appropriate and context-sensitive. This collaborative approach helps safeguard participant dignity and aligns with local norms and expectations.

### Participant safety

Participant safety and welfare will be prioritised throughout the study. All field researchers will be trained in standard operating procedures for participant safety, confidentiality, informed consent, and management of adverse events identified during data collection. Participants will receive feedback on selected health assessments, including BP, blood glucose, and anthropometric measurements.

Participants found to have elevated blood pressure, raised blood glucose, or other clinically significant findings that are previously undiagnosed or inadequately controlled will be informed of their results and advised to seek further assessment and treatment from appropriate healthcare providers. Referrals will be facilitated through local health services and community health workers in study sites where formal collaborations and referral agreements have been established during the site selection process. The study team will not provide diagnosis and treatment but will support referral and follow-up as appropriate.

A mental health risk management protocol will be implemented for participants reporting suicidal ideation, suicidal intent, or other indicators of significant psychological distress. Field researcher will be trained to recognise and respond to potential risks. Participants identified as being at risk will be referred immediately to designated healthcare professionals or appropriate mental health services according to local protocols. Where there is an immediate risk of self-harm, urgent referral procedures will be activated, and appropriate support persons may be engaged with the participant’s permission or where required to ensure safety.

Any adverse events or participant safety concerns arising during data collection will be documented, reviewed by the local study team, and managed according to study standard operating procedures and local ethical requirements. Participant confidentiality will be maintained throughout all referral and follow-up procedures.

### Data management

Data will be collected using REDCap (Research Electronic Data Capture), a secure, web-based data management platform, and stored on encrypted, password-protected servers. Each participant will be assigned a unique study identification number at enrolment. This identifier will be used to link data collected across all follow-up rounds while maintaining participant confidentiality. Participant contact information, linked to a unique study identification number, will be stored separately from study data and updated during follow-up visits to facilitate participant tracking and retention. Participant ID codes will anonymise records. Identifiable data (e.g., names, addresses) will be stored separately from study data under restricted access. Data will be stored on encrypted, password-protected servers hosted by the coordinating institution and managed in accordance with institutional data governance and data protection policies and the requirements of the UK International Data Transfer Agreement (IDTA) [24]. Participant contact information and other identifiable data (e.g., names and addresses) will be stored separately from study data under restricted access. Access to identifiable information will be limited to authorised members of the study team responsible for participant recruitment, follow-up, and data management, while de-identified datasets will be accessible only to approved investigators for analysis. Study findings will be shared with participating study communities, relevant public health authorities, policymakers, and stakeholders in participating countries through reports, policy briefs, presentations, and peer-reviewed publications. De-identified and aggregated data may be shared with public health agencies and collaborating institutions in accordance with ethical approvals, data-sharing agreements, and applicable national data protection regulations.

To ensure data quality, standardized data collection protocols will be used across all sites, supported by comprehensive training of field researcher. Built-in validation checks within REDCap will minimize entry errors, and real-time data monitoring dashboards will allow supervisors to track completeness and flag inconsistencies. Regular data audits, spot checks, and refresher training sessions will be conducted to reinforce protocol adherence and maintain high data integrity throughout the study.

To ensure comparability across countries, all questionnaires will undergo translation and back-translation, followed by pilot testing and cultural adaptation where required. Field researchers will receive standardised training prior to data collection, with periodic refresher training conducted throughout the study. Equipment will undergo regular calibration and maintenance according to predefined schedules. In addition, supervisory visits and routine quality assurance checks will be undertaken to maintain consistency, accuracy, and data quality across study sites.

### Data analysis

The analysis will aim to:

1. describe the period prevalence and co-occurrence of NCDs and their risk factors over a 24-month period;
2. explore causal relationships using multivariable models guided by directed acyclic graph (DAG) (Fig 1) by accounting for confounding, mediation and other possible effects;
3. assess longitudinal changes in risk factors and disease outcomes over time by mixed-effects regression models in a multilevel framework, with repeated measurements (Level 1) nested within individuals (Level 2) and individuals nested within households (Level 3) to account for clustering.

**Fig 1:**
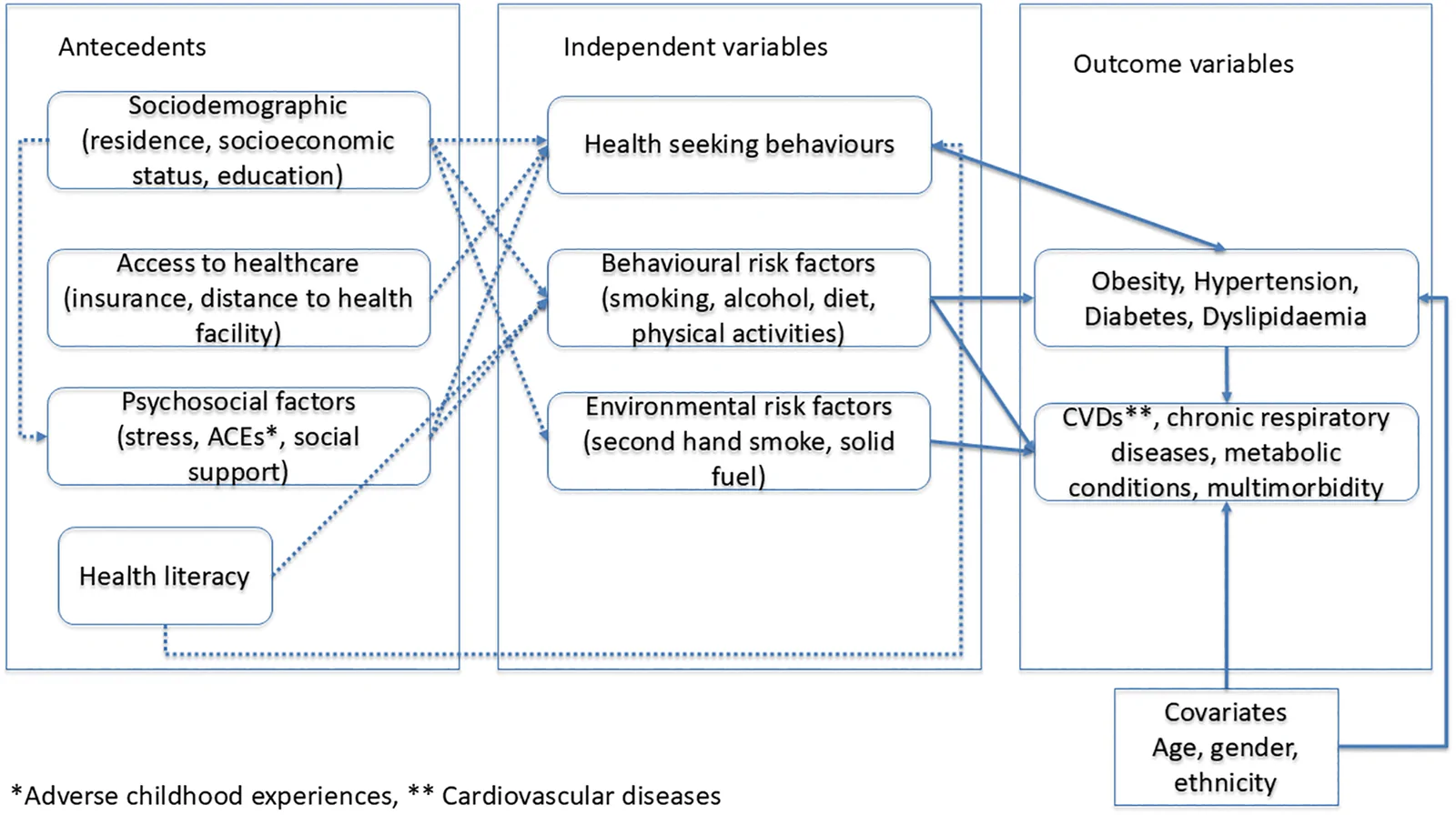
Directed acyclic graph of risk factors for non-communicable diseases (NCDs) outcomes.

Data will be checked and cleaned prior to analysis. The extent, pattern and type of any missing or invalid data will be assessed, and appropriate strategies (e.g., complete case analysis, multiple imputation) will be implemented as necessary. The sample will be summarised descriptively and analysed using a random intercepts hierarchical multiple logistic regression model, with the model hierarchy as above, incorporating fixed and random effects at model levels. The need for each level of the model will be verified by evaluation of variance partition coefficients in null models, with any level associated with negligible residual variance removed from the model. The selection of variables in the model will be guided by the findings of the DAG generated using theory and evidence and tested for goodness-of-fit against relevant metrics, to ensure that confounding, mediating and colliding variables are treated appropriately.

The analysis will also adopt an intersectional perspective, recognising that NCD risk and outcomes may be influenced by the interaction of multiple social, demographic, behavioural, and environmental factors [25]. Where appropriate, interaction terms and stratified analyses will be used to examine how characteristics such as age, sex, socioeconomic status, education, occupation, and place of residence intersect to influence NCD risk factors, multimorbidity, disease progression, healthcare utilisation, and health outcomes. These analyses will be guided by the use of a DAG for conceptual framework (Fig 1).

## Discussion

This study will contribute to advancing understanding of the growing burden of NCDs in Ghana, India and Nepal. While national surveys such as WHO STEPS, NFHS-5, and LASI have provided valuable prevalence estimates, their cross-sectional nature limits the ability to examine disease progression and transitions in risk exposure [2,7,8].

The proposed longitudinal cohort study fills this gap by enabling dynamic, life course analysis of NCD development across diverse sociocultural, economic and environmental contexts. Guided by DAG (Fig 1), the study conceptualises NCD outcomes including cardiovascular diseases, chronic respiratory diseases, metabolic conditions, multimorbidity, mental health disorders and dementia, as the result of complex interactions between upstream determinants (e.g., socioeconomic status, education, health literacy, access to care), intermediate exposures (e.g., tobacco and alcohol use, poor diet, physical inactivity, household air pollution), and psychosocial factors (e.g., stress and social support) [3,26].

A distinctive strength of the study is its household-based design, which enables examination of family- and household-level influences on NCD risk and outcomes that are often overlooked in individual-level analyses. Growing evidence suggests that NCD risk factors and chronic conditions cluster within households because family members share socioeconomic circumstances, dietary practices, physical activity patterns, environmental exposures, healthcare access, and health-related behaviours [27,28]. These factors are further shaped by household and community cultural norms, which may influence dietary choices, physical activity, tobacco and alcohol use, health-seeking behaviours, and perceptions of health and body weight [27,28]. Household-level social determinants, including income, household composition, and living conditions, have also been associated with multimorbidity and chronic disease burden [27,28]. Recent evidence from India demonstrated substantial household clustering of hypertension and diabetes, with nearly half of hypertension cases and over one-third of diabetes cases occurring in households where multiple members were affected [29]. This approach is particularly relevant in LMICs, where household dynamics, social support, caregiving, and collective decision-making play a significant role in shaping health behaviours and outcomes. By enrolling all eligible household members and collecting repeated longitudinal data, the study will enable investigation of shared environmental and behavioural exposures, intra-household variation in risk factors and outcomes, and the extent to which household-level factors contribute to NCD risk, multimorbidity, and disease progression beyond individual-level characteristics. The multilevel analytical framework will allow repeated measurements to be nested within individuals and individuals within households, enabling estimation of both individual- and household-level effects. Using a harmonised study tool, adapted from the WHO STEPS framework and expanded to include modules on mental health and household energy use, ensures that data collection is both comprehensive and comparable across sites. This facilitates cross-country analysis while allowing for contextual adaptation, thereby enhancing both scientific rigour and local relevance.

As with other population-based cohort studies, the potential for age- and sex-related recruitment bias should be recognised. Older adults with mobility limitations, multimorbidity, cognitive impairment, or other health-related barriers may be less likely to participate, potentially leading to underrepresentation of individuals with a higher NCD burden. Similarly, working-age adults may be more difficult to recruit because of employment, migration, commuting, or other competing commitments, while participation may differ by sex due to variations in employment patterns, caregiving responsibilities, and sociocultural factors. Such differential participation may influence estimates of NCD prevalence, risk factor distributions, and healthcare utilisation [30–32]. To minimise these risks, the study will employ repeat household visits, flexible scheduling, community engagement strategies, and ongoing monitoring of recruitment and retention patterns by age and sex. Findings will be interpreted in light of any observed differential participation.

Some methodological considerations should be taken into account when interpreting the findings of this study. First, behavioural risk factors and some health outcomes will be assessed through self-report and may therefore be subject to recall and social desirability biases. Participants may underreport behaviours perceived as socially undesirable, such as tobacco use, alcohol consumption, or mental health symptoms, and overreport healthy behaviours such as physical activity. To minimise these biases, data will be collected using standardised questionnaires administered by trained field researchers in a confidential setting. Second, differential participation and loss to follow-up may introduce selection bias if participation varies according to age, sex, socioeconomic status, health status, or mobility. Third, although objective measurements such as blood pressure, anthropometry, and blood glucose will be collected, some exposures and conditions cannot be measured comprehensively, and residual confounding may persist despite the use of longitudinal data and theory-informed analytical approaches. Finally, the cohort includes participants from selected catchment areas that were chosen to reflect a broad range of demographic, socioeconomic, environmental, and health-system contexts across the three countries. This diversity strengthens the external validity of the study and supports the relevance of findings across a range of comparable LMIC populations and settings. Analytical approaches such as weighting and statistical adjustment will be applied, where supported by available population data, to account for differences between study populations and wider target populations. These considerations will inform the interpretation of findings and their policy implications.

Operational challenges, such as participant mobility and retention, are anticipated and will be addressed proactively through a combination of community engagement, flexible follow-up strategies including home visits and phone interviews, and integration with local health systems. These measures are designed not only to improve data quality but also to foster trust and sustained participation among study communities. Importantly, the study is not only a scientific contribution but also a policy-oriented initiative. By identifying modifiable risk factors and vulnerable population groups, it will generate actionable evidence to inform targeted interventions and support national efforts toward UHC. Its alignment with SDG 3.4, which aims to reduce premature mortality from NCDs and promote mental health and well-being by 2030, positions the study as a strategic resource for governments, donors, and global health agencies seeking to address the NCD burden in a holistic and equitable manner.

## Conclusion

This multi-country study adopts a context-sensitive and methodologically rigorous approach to understanding NCD risk across diverse settings in Ghana, India, and Nepal. By integrating community-based sampling, secure digital data collection, and robust quality control measures, the study aims to generate high-quality, generalisable evidence to inform national and regional NCD strategies. Its design balances local relevance with broader applicability, contributing to the global effort to reduce the burden of NCDs through equitable, data-driven policy and programming.

## Supporting information

S1 File PLOS Inclusivity in Global Research Questionnaire. (DOCX)

## Abbreviations

DHS: Demographic and Health Surveys
GSHS: Global School-based Student Health Survey
LASI: Longitudinal Ageing Study in India
LMIC: low-and-middle-income countries
NCD: Non-communicable Disease
NNMS: National Noncommunicable Disease Monitoring Survey
NFHS: National Family Health Survey
OECD: Organisation for Economic Co-operation and Development
SDG: Sustainable Development Goal
UHC: Universal Health Coverage
WHO STEPS: World Health Organization STEPwise approach to NCD risk factor surveillance
