# Supplementary material for: A prospective longitudinal population-based cohort study of non-communicable diseases (NCDs) in Ghana, India, and Nepal (The GIN NCDs Study): A study protocol
Source: PLoS One. 2026 Nov 12;21(11):e0362114. doi: 10.1371/journal.pone.0362114 (PMC13759048; doi:10.1371/journal.pone.0362114)
Supplement: Supplementary file 1 [file pone.0362114.s001.docx]

Inclusivity in global research

PLOS’ policy on inclusivity in global research aims to improve transparency in the reporting of research performed outside of researchers’ own country or community and ensures that PLOS publications reporting global research adhere to high standards for research ethics and authorship. Authors of relevant research articles may be asked to complete the questionnaire below, which outlines ethical, cultural, and scientific considerations specific to inclusivity in global research. This questionnaire may be requested when researchers have travelled to a different country to conduct research, if research uses samples collected in another country, research with Indigenous populations or their lands, or if research is on cultural artefacts. Researchers travelling to another country solely to use laboratory equipment will not normally be required to complete the questionnaire. However, the questionnaire can be requested at the journal’s discretion for any submission – if you have been requested to complete this questionnaire by the PLOS journal you submitted to, please do so.

Please complete the questionnaire below and include this as a Supporting Information file with your manuscript. Note that if your paper is accepted for publication, this checklist will be published with your article in the supporting information files. Please ensure that you reference the checklist in the main body of your manuscript. We suggest adding a subsection ‘Inclusivity in global research’ to your Methods section and adding the following sentence: “Additional information regarding the ethical, cultural, and scientific considerations specific to inclusivity in global research is included in the Supporting Information (SX Checklist)”

The questions have been designed to be applicable to a wide range of study types, and there are subsections for both human subjects research and non-human subjects research. If any of the questions are not relevant to your research please mark them as “N/A” as appropriate.

**Ethical considerations, permits and authorship**

*This section is applicable to all research types.*

Provide details as to who granted permissions and/or consent for the study to take place in the Methods section of your manuscript. This should include the names of **all** ethics boards, governmental organizations, community leaders or other bodies that provided approval for the study. If individuals provided approval refer to these people by their role or title but do not list their name(s).

Reported on page number: Page 8

If there were any deviations from the study protocol after approval was obtained please provide details of these changes in the Methods section of your manuscript.
Did this study involve local collaborators that are residents of the country where the research was conducted or members of the community studied? If you do not have any authors from said communities, please provide an explanation for this below.

Reported on page number: no deviations from the study protocol after approval

Yes. The study includes local collaborators from all participating countries, including researchers from the University of Ghana (AEY, ENYN, EHO), Datta Meghe Institute of Higher Education & Research (MNK, AG, ZQ, ST), Green Tara Nepal (BK, RCS, AA, AS), and Tribhuvan University (RP). These collaborators are actively involved in study design, implementation, stakeholder engagement, and interpretation of findings, ensuring the research is locally informed and contextually relevant.

Everyone listed as an author should meet PLOS’ criteria for authorship and all individuals who meet these criteria should be included in the author byline, rather than the acknowledgements. For further information please see the journal’s Authorship Policy.

**Human subjects research (e.g. health research, medical research, cross-cultural psychology)**

Did you obtain written informed consent from a representative of the local community or region before the research took place? How did you establish who speaks for the community? Details of written informed consent obtained from study participants should be reported separately in the Methods section of your manuscript.

Yes. Prior to study implementation, permission and support were obtained from relevant local authorities and community stakeholders in each study site, including local government, health, and community leaders, as appropriate to the local context. These stakeholders were identified through engagement with local partner institutions and established administrative and community governance mechanisms. Written informed consent from individual study participants is described separately in the Methods section (Page 10-11) of the manuscript.

How did members of the local community provide input on the aims of the research investigation, its methodology, and its anticipated outcome(s)?

Local community input was incorporated through consultations with local partners, health authorities, and community stakeholders during study development. Their feedback informed the selection of research priorities, study procedures, recruitment approaches, and implementation strategies to ensure the study was contextually appropriate and responsive to local health needs. Ongoing engagement with these stakeholders will also support interpretation and dissemination of findings. (Page 6 of the manuscript)

When engaging with the local community, how did you ensure that the informed consent documents and other materials could be understood by local stakeholders?

Will the findings of the research be made available in an understandable format to stakeholders in the community where the study was conducted (e.g. via a presentation, summary report, copies of publications, etc.)? Please provide details of how this will be achieved.

Yes, findings will be disseminated to stakeholders at community, provincial, and national levels in each participating country through knowledge-sharing and engagement strategy. Community-level feedback meetings will be organised in study sites to present key findings in accessible, local, non-technical language. Plain-language summary reports, translated into relevant local languages, will be shared with community representatives, participants, local health facilities, and community leaders.

At the provincial and national levels, findings will be disseminated through stakeholder workshops, policy dialogues, and presentations involving Ministries of Health, public health agencies, and collaborating institutions in Ghana, India, and Nepal. Policy briefs highlighting key findings and implications for NCD prevention and control will be developed and shared with decision-makers. In addition, scientific outputs, including peer-reviewed publications and conference presentations, will be made openly accessible whenever possible.

Throughout the study, local implementing partners and community stakeholders will be engaged in interpreting and contextualising findings to ensure that results are relevant and meaningful to the populations involved. This dissemination approach aims to support local ownership of the evidence, inform public health planning, and contribute to the development of context-appropriate NCD policies and interventions. (Page 11 of manuscript)

To ensure that study materials were understandable and culturally appropriate, participant information sheets, consent forms, and data collection tools were translated into relevant local languages and reviewed by in-country research partners. Materials were pilot-tested and refined where necessary to improve clarity and comprehension. Trained field researchers were also available to explain the study, answer questions, and provide information in participants’ preferred language before consent was obtained. (Page 11 of manuscript)

**Non-human subjects research using specimens/ animals collected as part of the study, or those housed in archival collections. Examples include archaeology, paleontology, botany and zoology.**

Did the permission you obtained from a local authority to perform the study include an agreement on access to outputs and benefit sharing? This may include procedures to enable fair distribution of the benefits and resources arising from the research performed. Please include any details of Prior Informed Consent and Benefit Sharing Agreements obtained. These may be required by field-specific regulations, for example the Convention on Biological Diversity (CBD) and the associated Nagoya Protocol.

Not relevant

If the material used in your study was imported, please A) provide the year it was imported and B) indicate whether permits were obtained to import/export the materials used, C) provide details of any permits obtained. If this information is not available, please indicate this.

Not relevant

If you used archival specimens, please state how the material used in your study was acquired by the institute it is held in and provide details of any permits obtained for the original excavations/ sample collection. If this information is not available, please indicate this.

Not relevant

How was the potential cultural significance of the materials collected in your study to local communities considered in your research design? Were Indigenous peoples and/or local researchers and institutions involved with archaeological excavations / collection of specimens? If so, please provide a description of their involvement.

Not relevant

If your manuscript includes photographs of human remains please indicate whether authors obtained permission from descendants or affiliated cultural communities to do so.

Not relevant
