# Supplementary material for: A prospective longitudinal population-based cohort study of non-communicable diseases (NCDs) in Ghana, India, and Nepal (The GIN NCDs Study): A study protocol
Source: PLoS One. 2026 Nov 12;21(11):e0362114. doi: 10.1371/journal.pone.0362114 (PMC13759048; doi:10.1371/journal.pone.0362114)
Supplement: Supplementary file 2 [file pone.0362114.s002.docx]

Editorial Team

PLOS One

13^th^ October 2026

**Re: Manuscript reference number-PONE-D-26-31442**

Dear Editorial Team,

I am writing to submit a revision of the above manuscript. We sincerely appreciate the helpful comments from the reviewer, and we have revised the manuscript according to his/her suggestions.

The modified sections in the manuscript are highlighted using tracked changes. Please find the attached response to the reviewer which details the changes we have made along with explanations for these changes.

We hope that the revised manuscript will now be acceptable for publication in *PLOS One* and look forward to your decision. Thank you very much for your consideration of this manuscript.

Yours sincerely,

Dr Kaung Suu Lwin, MBBS MHSc PhD

School of Human and Health Sciences

University of Huddersfield | Queensgate | Huddersfield | HD1 3DH

**RESPONSE TO EDITOR**

*1) Please ensure that your manuscript meets PLOS ONE's style requirements, including those for file naming. The PLOS ONE style templates can be found at….*

Thank you, we have now reviewed the revised manuscript to comply with PLOS ONE requirements, including manuscript structure, section headings, references, tables, figures, and file naming. We have also checked the PLOS ONE formatting templates and updated the submission files accordingly.

*2) Please include a complete copy of PLOS’ questionnaire on inclusivity in global research in your revised manuscript. Our policy for research in this area …*

Thank you for highlighting this, we have now completed the PLOS Inclusivity in Global Research Questionnaire, and submitted it as **Supporting Information** and alongside the revised manuscript.

*3) Thank you for stating following financial disclosure: [This study is funded by university research fund from the University of Huddersfield]… state what role the funders took in the study…*

Thank you, we have revised the funding statement to clarify the role of the funder (the University of Huddersfield); however, the funder had no role in the design of the study, data collection, data analysis, interpretation of findings, decision to publish, or preparation of the manuscript. The following statement has been added to the cover letter and will be included in the funding information:

“This study is funded by the University Research Fund, University of Huddersfield. The funders had no role in study design, data collection and analysis, decision to publish, or preparation of the manuscript.”

*4) you indicated that you will make your data available on acceptance. We strongly recommend all authors decide on a data sharing plan before acceptance, as the process can be lengthy and hold up publication timelines. Please note that, though access restrictions are acceptable now, your entire data will need to be made freely accessible if your manuscript is accepted for publication. This policy applies to all data except where public deposition would breach compliance with the protocol approved by your research ethics board. If you are unable to adhere to our open data policy, please kindly revise your statement to explain your reasoning and we will seek the editor's input on an exemption...*

Thank you, while we support data sharing, our study collects detailed individual-level demographic, behavioural, health, and geographic data from participants in specific communities across Ghana, India, and Nepal. Given the longitudinal design and relatively small sampling clusters, there is a potential risk of participant re-identification even after de-identification. In addition, participants consented to data use under approved research governance arrangements and did not provide consent for unrestricted public data release.

We therefore request an exemption from unrestricted public deposition. De-identified data will be made available to qualified researchers upon reasonable request, subject to review to ensure compliance with participant consent, ethical approvals, and data protection requirements.

**“Data Availability Statement**

The data underlying this study cannot be publicly shared due to ethical and confidentiality restrictions. Because the dataset contains detailed individual-level health, demographic, and geographic information from participants in specific communities, there is a potential risk of re-identification. De-identified data will be made available to qualified researchers upon reasonable request, subject to approval and compliance with participant consent and ethical requirements.”

*5).Please amend either title on online submission form or in manuscript so that they are identical.*

Thank you. We have revised them to ensure they are identical.

*6) upload a copy of Supporting Information Figure/Table/etc. [S1 Fig. Directed acyclic graph of risk factors for non-communicable diseases (NCDs) outcomes.] which you refer to on page 12.*

Thank you for bringing this to our attention. We apologise for the inconsistency. The directed acyclic graph (DAG) is intended to be presented as Figure 1 in the main manuscript, not as Supporting Information. We have corrected the manuscript text and figure citations accordingly.

*7) Please include captions for your Supporting Information files at the end of your manuscript, and update any in-text citations to match accordingly. Please see our Supporting Information guidelines for more information:* [*http://journals.plos.org/plosone/s/supporting-information*](http://journals.plos.org/plosone/s/supporting-information)*.*

We have added captions for all Supporting Info. files at the end of manuscript and updated the corresponding in-text citations.

*8).If reviewer comments include a recommendation to cite specific previously published works, please evaluate these publications to determine whether they are relevant and should be cited. There is no requirement to cite these works unless the editor has indicated otherwise.*

Thank you, but no reviewer requested the inclusion of additional citations. However, we reviewed the manuscript to ensure that all references are relevant, current, and appropriate to the study.

**RESPONSE TO REVIEWERS**

We would like to thank the editor and reviewer for their helpful comments. Our responses are given beneath each comment. The added/revised text is double-quoted in our response for ease of reference, with line and page numbers provided where necessary.

*Reviewer #1:*

1) *Introduction: Why did you choose Ghana, India and Nepal out of all LMICs?*

Thank you. We purposefully selected Ghana, India, and Nepal to capture diversity in NCD epidemiology, demographic transition, urbanisation, socioeconomic development, and health-system capacity across LMIC settings. Together, these countries represent two major LMIC regions, South Asia and sub-Saharan Africa, allowing the study to examine both common and context-specific determinants of NCDs across diverse populations and environments.

The selected countries face a substantial and growing burden of NCDs, driven by rapid urbanisation, population ageing, and lifestyle changes, yet there remains a lack of comparable longitudinal population-based evidence to understand disease progression, multimorbidity, and changes in risk factors over time. Including countries with differing demographic profiles, cultural contexts, and healthcare systems enhances the opportunity to generate evidence that is both locally relevant and broadly informative for other LMIC settings.

In addition, country selection was guided by practical considerations essential for establishing a long-term cohort study, including the availability of strong local research partnerships, existing field infrastructure, experienced research teams, and institutional commitment to support participant follow-up and high-quality data collection over time.

We acknowledge that Ghana, India, and Nepal are not intended to be representative of all LMICs. Rather, they were selected to provide a range of contrasting contexts that can improve understanding of NCD trajectories and determinants across diverse low- and middle-income settings.

Revision made in the manuscript:

“Most existing national health datasets in Ghana, India, and Nepal are cross-sectional, limiting their ability to examine disease progression, changes in risk exposure, and intergenerational health patterns over time. Longitudinal evidence is needed to better understand how NCDs develop, cluster, and interact across the life course within diverse sociocultural and economic contexts. Ghana, India, and Nepal were selected to capture variation in NCD epidemiology, demographic transition, urbanisation, socioeconomic development, and health-system capacity across LMIC settings. Although the countries differ in demographic characteristics, health-system contexts, and stages of epidemiological transition, they share a rapidly increasing NCD burden driven by common behavioural and metabolic risk factors, including unhealthy diets, physical inactivity, tobacco use, obesity, hypertension, and diabetes [2,6,10]. Studying these settings using harmonised methods will enable comparison of both shared and context-specific determinants of NCDs, helping to identify factors that are consistent across populations as well as those shaped by local social, environmental, and health-system contexts. Although not intended to be representative of all LMICs, the inclusion of countries from South Asia and sub-Saharan Africa provides an opportunity to generate transferable evidence on NCD prevention and control across diverse settings. The countries were also selected based on practical considerations essential for establishing a sustainable longitudinal cohort, including the availability of strong local research partnerships, existing field infrastructure, experienced research teams, and institutional commitment to support long-term participant follow-up and high-quality data collection.” (Page 3-4, Lines 87-104)

2) *What are similarities and differences in risk factors and NCDs among them? Will you be comparing the results across these countries? Why do you think they should be compared?*

Thank you for this insightful comment. Although the three countries differ in demographic profiles, levels of urbanisation, socioeconomic development, health-system capacity, and stages of epidemiological transition, they share a rapidly growing burden of NCDs driven by common behavioural and metabolic risk factors, including unhealthy diets, physical inactivity, tobacco use, obesity, hypertension, and diabetes. The study will use harmonised methods across all sites, enabling both within-country and cross-country analyses. Comparing these diverse settings will help identify determinants and disease patterns that are shared across populations as well as those that are context-specific, thereby generating evidence that is both locally relevant and transferable to other LMIC settings. In addition, the selection of these countries was informed by practical considerations essential for establishing a long-term cohort study, including the availability of strong local research partnerships, existing field infrastructure, experienced research teams, and institutional commitment to support participant follow-up and high-quality data collection over time. We have revised the manuscript accordingly. Please see the revised text reported in response to Comment 1.

*Methodology:*

3) *When reading about India, I think of two big topics related to NCDs are air pollution and rich-poor gap among populations. Do other two countries have similar backgrounds?*

Thank you for this insightful comment. We agree that air pollution and socioeconomic inequalities are important drivers of NCDs in India. Similar challenges are also present in Ghana and Nepal, although their magnitude and underlying drivers differ across settings. Air pollution contributes substantially to cardiovascular and respiratory disease in all three countries, while socioeconomic inequalities influence exposure to NCD risk factors, healthcare access, diagnosis, treatment, and health outcomes. These shared and contrasting contextual factors strengthen the rationale for cross-country comparisons and provide an opportunity to identify both common and context-specific determinants of NCDs using harmonised longitudinal data. To clarify this, we have added the following to the Background and Rationale section.

Revision made in the manuscript:

“Beyond behavioural and metabolic risk factors, Ghana, India, and Nepal are also affected by broader environmental and social determinants of NCDs. Air pollution has emerged as an important contributor to cardiovascular and respiratory disease across all three countries. In Nepal, exposure to ambient and household air pollution is associated with substantial burdens of ischaemic heart disease, stroke, chronic respiratory disease, and lung cancer, while air pollution in Ghana contributes to significant cardiovascular and respiratory morbidity and mortality [11,12]. In India, air pollution is increasingly recognised as a major contributor to cardiovascular and respiratory diseases and acts alongside rapid urbanisation and lifestyle changes to accelerate the NCD burden [13]. Socioeconomic inequalities also play an important role in shaping NCD risk and outcomes across the three countries. Evidence from India, Ghana, and Nepal demonstrates disparities in exposure to risk factors, healthcare access, diagnosis, treatment, and health outcomes according to wealth, education, place of residence, and other social determinants [14-16]. These shared challenges, together with differences in environmental exposures, patterns of urbanisation, and health-system contexts, provide a valuable opportunity to investigate both common and context-specific determinants of NCDs using harmonised longitudinal data.” (Page 4, Lines 105-119)

4) *I believe air pollution is an exposure in your study with risk factors of secondhand smoke and solid fuel. Did you also take account of outdoor air pollution?*

We agree that outdoor air pollution is an important risk factor for NCDs, particularly cardiovascular and respiratory diseases. In the current study, we will collect household-level information on exposures related to indoor air pollution, including second-hand smoke exposure and the use of solid fuels for cooking and heating. However, direct measurement of outdoor air pollution is beyond the scope of the baseline data collection. Where feasible, we plan to explore links between participants' residential locations and publicly available environmental and geospatial datasets, including ambient air pollution estimates, in future analyses.

5) *Line 132 and 133: These sites have been chosen to reflect a range of geographic, socioeconomic, and health system contexts, enabling the study to capture variation in exposure to risk factors and access to care. I would love to know more about socioeconomic considerations and which areas were selected in each country to account for it.*

Thank you for this comment. We agree that the socioeconomic rationale for site selection was not sufficiently described in the manuscript. In addition to geographic diversity, the selected sites were chosen to capture variation in socioeconomic conditions, levels of urbanisation, environmental exposures, and healthcare access within each country. In Ghana, Ga East Municipality in the Greater Accra region include rapidly urbanising and peri-urban areas with diverse socioeconomic profiles and varying access to health services. In Nepal, Nagarjun Municipality comprises communities with differing socioeconomic characteristics and levels of urban development. In India, Hingini Block in Wardha District, Maharashtra, was selected because it encompasses a mix of urban, peri-urban, and rural communities undergoing demographic and socioeconomic transition. Collectively, these sites provide an opportunity to examine how socioeconomic and health-system factors influence NCD risk, disease progression, multimorbidity, and healthcare utilisation across diverse LMIC contexts. We have revised the manuscript to clarify these considerations.

Revision made in the manuscript:

“In Ghana, the study will be conducted in Ga East Municipality in the Greater Accra region that represent a range of peri-urban and rapidly developing settings with varying socioeconomic profiles and healthcare access. In India, the study will be conducted in Wardha and Seloo blocks of Wardha District, Maharashtra, which encompass urban, peri-urban, and rural communities with varying socioeconomic characteristics and access to healthcare services. In Nepal, the study will be conducted in Nagarjun Municipality, Kathmandu District, Bagmati Province, which includes communities with diverse socioeconomic characteristics and varying levels of urbanisation and service availability. Together, these sites provide an opportunity to examine how social, environmental, and health-system factors shape NCD risk, progression, multimorbidity, and access to care across different population groups.” (Page 5, Lines 166-175)

6) *What age‑group categories are you planning to use in the study?*

Thank you for this question. During the initial phase, the study will enrol adults aged 18 years and above. For analysis and reporting, participants will be grouped into the following age categories: 18-29, 30-44, 45-59, and ≥60 years. These categories will be selected to represent key stages of adulthood and to facilitate the assessment of age-related differences in NCD risk factors, disease prevalence, multimorbidity, and healthcare utilisation. Additional age categorizations may be applied for specific analyses where appropriate. We have clarified this in the manuscript.

Revision made in the manuscript:

“For analysis and reporting purposes, participants will be grouped into the following age categories: 18-29, 30-44, 45-59, and ≥60 years. These categories reflect key stages of adulthood and will facilitate the assessment of age-related differences in NCD risk factors, disease prevalence, multimorbidity, and healthcare utilisation. Additional age categorizations may be used for specific analyses where appropriate. ” (Page 6, Lines 178-182)

7) *Will individuals with existing diseases be included?*

Thank you, yes, individuals with existing NCDs will be eligible for inclusion, provided they meet the study eligibility criteria and are able to provide informed consent. The cohort is intended to capture the full spectrum of NCD risk and disease, including both individuals with and without pre-existing conditions. Including participants with established NCDs will enable the study to examine disease progression, multimorbidity, treatment patterns, healthcare utilisation, and long-term outcomes, while also allowing comparisons with individuals who are disease-free at baseline. We have clarified this in the manuscript.

Revision made in the manuscript:

“Initially, all residents in study areas aged 18 years and above will be eligible to participate, regardless of their NCD status, with plans to gradually include individuals of all ages.” (Page 5, Lines 175-176)

8) *How do you make sure that you are capturing related risk factors for each disease? For example, does your mental health outcome fully capture the spectrum of mental health disorders when assessed only through the WHO STEPS depression and suicide modules? Do you have subject matter experts for specific diseases who can advise?*

Thank you for this important comment. We acknowledge that no single survey instrument can comprehensively capture the full spectrum of risk factors and outcomes across all NCDs. The study adopts a pragmatic approach by using the WHO STEPS framework as the core platform because it provides standardized and internationally comparable measures of major behavioural and metabolic risk factors. For condition-specific outcomes, we will supplement the core STEPS modules with additional measures where appropriate and feasible.

We agree that the WHO STEPS depression and suicide modules do not capture the full range of mental health disorders. Our intention is to assess common symptoms of depression and suicidal behaviour as key mental health indicators rather than provide comprehensive psychiatric diagnoses. The study is designed primarily for population-level surveillance and longitudinal assessment of NCD risk factors and outcomes rather than clinical diagnosis of specific disorders. We will therefore interpret mental health findings within these limitations.

In addition, the study team includes investigators and collaborators with expertise across multiple disciplines, including cardiovascular disease, diabetes, mental health, epidemiology, public health, implementation science, biostatistics, and health systems research. Disease-specific experts will contribute to developing study instruments, interpreting findings, and refining future data collection waves to ensure important disease-specific risk factors and outcomes are appropriately captured. We have clarified this in the manuscript.

Revision made in the manuscript:

“The questionnaires' development and data interpretation will be informed by multidisciplinary subject matter experts with experience in cardiovascular disease, diabetes, mental health, epidemiology, public health, and health systems research.” (Page 8, Lines 261-263)

“These measures are intended to identify common mental health symptoms and population-level patterns rather than provide comprehensive diagnostic assessment of mental health disorders.” (Page 9, Lines 288-290)

9) *Do you also take account of intersectional considerations between risk factors and NCDs?*

Yes, our study recognises that NCD risk and outcomes are shaped by the intersection of multiple social, demographic, behavioural, and environmental factors. The collection of detailed information on age, sex, education, occupation, socioeconomic status, place of residence, lifestyle behaviours, environmental exposures, and healthcare access will enable investigation of how these factors interact to influence NCD risk, multimorbidity, disease progression, and health outcomes. In addition to the planned multivariable analyses, we will explore effect modification and interaction effects where theoretically and empirically justified. We have revised the Data Analysis section to clarify this approach.

Revision made in the manuscript:

“The analysis will also adopt an intersectional perspective, recognising that NCD risk and outcomes may be influenced by the interaction of multiple social, demographic, behavioural, and environmental factors [25]. Where appropriate, interaction terms and stratified analyses will be used to examine how characteristics such as age, sex, socioeconomic status, education, occupation, and place of residence intersect to influence NCD risk factors, multimorbidity, disease progression, healthcare utilisation, and health outcomes. These analyses will be guided by the use of a DAG for conceptual framework (Figure 1).” (Page 12, Lines 413-419)

10) *Will there be a benefit or incentive for participants? Could this affect the study in any way?*

Thank you for this question. Participants will not receive direct financial incentives, however, they will benefit from free health assessments conducted as part of the study, including measurements of blood pressure, anthropometry, and blood glucose, as well as feedback on key findings and referral to appropriate health services when clinically significant abnormalities are identified. We believe that these benefits may enhance participant engagement and retention while minimising financial inducement. As the assessments are offered uniformly to all eligible participants, we do not anticipate that they will substantially influence study outcomes. Nevertheless, we acknowledge that increased awareness of health status may influence subsequent health-seeking behaviour and risk-factor modification, which will be considered when interpreting longitudinal findings. We have clarified this in the manuscript.

Revision made in the manuscript:

“Participants will not receive financial incentives for participation. As a benefit of participation, individuals will receive feedback on selected health assessments, including anthropometric measurements, blood pressure and blood glucose measurements, and will be referred to appropriate healthcare services when clinically significant findings are identified. These procedures are intended to promote ethical conduct and participant welfare while minimising undue influence on participation.” (Page 10, Lines 328-333)

11) *What procedures are in place for participants who are found to have elevated blood pressure or raised blood glucose during a measurement but are not currently receiving treatment? Additionally, how will cases of suicidal ideation identified through the survey be managed?*

Thank you. Participants found to have elevated blood pressure or raised blood glucose during study assessments will be informed of their results and advised to seek further evaluation and management at an appropriate healthcare facility. Referrals will be facilitated through local health services and community health workers in study sites where formal collaborations and referral agreements have been established during the site selection process. The study team will not provide clinical treatment but will implement a referral pathway to ensure participants with abnormal findings receive appropriate follow-up care.

For mental health, participants reporting suicidal ideation or intent will be managed according to a predefined risk management protocol. Trained field researcher will immediately notify the site supervisor, who will conduct an initial risk assessment and facilitate urgent referral to appropriate mental health or emergency services in accordance with local procedures. Information on available support services will be provided, and participant safety and confidentiality will be prioritised throughout. We have clarified these procedures in the manuscript as follows:

“Participant safety

Participant safety and welfare will be prioritised throughout the study. All field researchers will be trained in standard operating procedures for participant safety, confidentiality, informed consent, and management of adverse events identified during data collection. Participants will receive feedback on selected health assessments, including BP, blood glucose, and anthropometric measurements.

Participants found to have elevated blood pressure, raised blood glucose, or other clinically significant findings that are previously undiagnosed or inadequately controlled will be informed of their results and advised to seek further assessment and treatment from appropriate healthcare providers. Referrals will be facilitated through local health services and community health workers in study sites where formal collaborations and referral agreements have been established during the site selection process. The study team will not provide diagnosis and treatment but will support referral and follow-up as appropriate.

A mental health risk management protocol will be implemented for participants reporting suicidal ideation, suicidal intent, or other indicators of significant psychological distress. Field researcher will be trained to recognise and respond to potential risks. Participants identified as being at risk will be referred immediately to designated healthcare professionals or appropriate mental health services according to local protocols. Where there is an immediate risk of self-harm, urgent referral procedures will be activated, and appropriate support persons may be engaged with the participant's permission or where required to ensure safety.

Any adverse events or participant safety concerns arising during data collection will be documented, reviewed by the local study team, and managed according to study standard operating procedures and local ethical requirements. Participant confidentiality will be maintained throughout all referral and follow-up procedures.” (Page 10, Lines 340-362)

12) *Discussion: What age and sex related recruitment biases do you anticipate, and how might these influence the study findings?*

Thank you for this important comment. We acknowledge that age- and sex-related recruitment biases may occur in population-based cohort studies. Older adults with mobility limitations, severe illness, cognitive impairment, or caregiving responsibilities may be less likely to participate, potentially resulting in underrepresentation of individuals with a higher burden of NCDs. Recruitment challenges may also occur among working-age adults, particularly those engaged in full-time employment, shift work, migration, or occupations requiring long periods away from home. In addition, sex-related differences in participation may arise because men are often absent during daytime household visits due to employment, while women may face competing domestic responsibilities or sociocultural barriers to participation in some settings. These patterns could influence estimates of disease prevalence, risk factor distributions, and healthcare utilisation.

To minimise these biases, recruitment will include repeat household visits at different times of the day and week, flexible scheduling, and engagement with local community leaders and health workers. Recruitment and retention patterns will be monitored by age and sex throughout the study, and potential selection biases will be assessed and accounted for in analyses where appropriate. We have clarified this in the revised manuscript:

“As with other population-based cohort studies, the potential for age- and sex-related recruitment bias should be recognised. Older adults with mobility limitations, multimorbidity, cognitive impairment, or other health-related barriers may be less likely to participate, potentially leading to underrepresentation of individuals with a higher NCD burden. Similarly, working-age adults may be more difficult to recruit because of employment, migration, commuting, or other competing commitments, while participation may differ by sex due to variations in employment patterns, caregiving responsibilities, and sociocultural factors. Such differential participation may influence estimates of NCD prevalence, risk factor distributions, and healthcare utilisation [30-32]. To minimise these risks, the study will employ repeat household visits, flexible scheduling, community engagement strategies, and ongoing monitoring of recruitment and retention patterns by age and sex. Findings will be interpreted in light of any observed differential participation.” (Page 13, Lines 458-468)

13) *Any other limitations and potential bias that should be taken into consideration in interpreting results… Could there be social desirability bias due to participants self‑reporting risk factors?*

Thank you for this important comment. We agree that we should consider potential sources of bias when interpreting study findings. Self-reported measures of behavioural risk factors, including tobacco use, alcohol consumption, dietary practices, physical activity, and mental health symptoms, may be subject to recall and social desirability biases, potentially leading to misclassification of exposures. Selection bias may also arise through differential participation or loss to follow-up, particularly among older adults, working-age individuals, and highly mobile populations. In addition, residual confounding may remain despite the use of theory-driven analytical approaches and longitudinal data. Finally, although the study is conducted in selected catchment areas, these areas were purposefully chosen to reflect diverse demographic, socioeconomic, environmental, and health-system contexts across the three countries. This diversity strengthens the external validity of the study and enhances the relevance of the findings across a range of comparable LMIC populations and settings. Analytical approaches such as weighting and statistical adjustment will be applied, where supported by available population data, to account for differences between study populations and wider target populations. We have added these considerations to the Discussion section, which now reads:

“Some methodological considerations should be taken into account when interpreting the findings of this study. First, behavioural risk factors and some health outcomes will be assessed through self-report and may therefore be subject to recall and social desirability biases. Participants may underreport behaviours perceived as socially undesirable, such as tobacco use, alcohol consumption, or mental health symptoms, and overreport healthy behaviours such as physical activity. To minimise these biases, data will be collected using standardised questionnaires administered by trained field researchers in a confidential setting. Second, differential participation and loss to follow-up may introduce selection bias if participation varies according to age, sex, socioeconomic status, health status, or mobility. Third, although objective measurements such as blood pressure, anthropometry, and blood glucose will be collected, some exposures and conditions cannot be measured comprehensively, and residual confounding may persist despite the use of longitudinal data and theory-informed analytical approaches. Finally, the cohort includes participants from selected catchment areas that were chosen to reflect a broad range of demographic, socioeconomic, environmental, and health-system contexts across the three countries. This diversity strengthens the external validity of the study and supports the relevance of findings across a range of comparable LMIC populations and settings. Analytical approaches such as weighting and statistical adjustment will be applied, where supported by available population data, to account for differences between study populations and wider target populations. These considerations will inform the interpretation of findings and their policy implications.(Page 13, 469-487)

14) *It was said that a key strength of the study is its household based design, which allows for the examination of shared exposures and family level influences. This appears to be a distinctive and potentially novel aspect. It would be helpful to expand on this component by providing more background from the literature on household level determinants of NCDs and explaining how you plan to analyze these relationships. Additionally, outlining the specific hypotheses related to household clustering, shared environmental or behavioral exposures, and intra family variation in risk factors or outcomes would strengthen this section.*

Thank you for this positive comment. We agree that the household-based design represents a distinctive strength of the study and merits further explanation. Household environments influence NCD risk through shared socioeconomic conditions, dietary practices, physical activity patterns, health-related behaviours, environmental exposures, healthcare access, and health literacy. Family members may also influence each other's health behaviours through social norms, caregiving, and shared decision-making. By enrolling all eligible household members and following them longitudinally, the study will provide a unique opportunity to examine both individual- and household-level determinants of NCDs.

To address this, we have expanded the Discussion section to describe the importance of household-level influences on NCDs and to clarify our analytical approach. Specifically, we will use multilevel models with individuals nested within households to quantify household clustering of NCD risk factors and outcomes. We will also examine associations between household-level characteristics and individual health outcomes. We have further clarified our hypotheses regarding shared environmental and behavioural exposures, household clustering of NCD risk factors, and variation in outcomes within and between households.

Revision made in the manuscript:

“A distinctive strength of the study is its household-based design, which enables examination of family- and household-level influences on NCD risk and outcomes that are often overlooked in individual-level analyses. Growing evidence suggests that NCD risk factors and chronic conditions cluster within households because family members share socioeconomic circumstances, dietary practices, physical activity patterns, environmental exposures, healthcare access, and health-related behaviours [27.28]. These factors are further shaped by household and community cultural norms, which may influence dietary choices, physical activity, tobacco and alcohol use, health-seeking behaviours, and perceptions of health and body weight [27,28]. Household-level social determinants, including income, household composition, and living conditions, have also been associated with multimorbidity and chronic disease burden [27,28]. Recent evidence from India demonstrated substantial household clustering of hypertension and diabetes, with nearly half of hypertension cases and over one-third of diabetes cases occurring in households where multiple members were affected [29]. This approach is particularly relevant in LMICs, where household dynamics, social support, caregiving, and collective decision-making play a significant role in shaping health behaviours and outcomes. By enrolling all eligible household members and collecting repeated longitudinal data, the study will enable investigation of shared environmental and behavioural exposures, intra-household variation in risk factors and outcomes, and the extent to which household-level factors contribute to NCD risk, multimorbidity, and disease progression beyond individual-level characteristics. The multilevel analytical framework will allow repeated measurements to be nested within individuals and individuals within households, enabling estimation of both individual- and household-level effects. Using a harmonised study tool, adapted from the WHO STEPS framework and expanded to include modules on mental health and household energy use, ensures that data collection is both comprehensive and comparable across sites. This facilitates cross-country analysis while allowing for contextual adaptation, thereby enhancing both scientific rigour and local relevance.” (Page 12-13, Lines 434-457)

15) *Reviewer #2: I read with interest the manuscript ..Line 45 - This study will generate longitudinal evidence on NCD risk factors, multimorbidity, and mental health in LMICs, addressing critical data gaps. Lines 116-117 At Year 0 (Baseline), a comprehensive assessment will be conducted, including demographic, 116 behavioural, clinical, and anthropometric measurements. Need to specify what the clinical parameters would be.*

Thank you for this comment. We agree that “clinical measurements” was inappropriate. We have revised the text to refer to “physical and anthropometric measurements”. These include blood pressure, heart rate, blood glucose, peak expiratory flow, height, weight, waist circumference, and hip circumference. Detailed descriptions of these measurements are provided in the health outcomes and measurements section of the manuscript.

Revision made in the manuscript:

“At Year 0 (Baseline), a comprehensive assessment will be conducted, including demographic, behavioural, physical, and anthropometric measurements.” (Page 5, Lines 148-149)

16) *Lines 158-9 -While the study is conducted within defined catchment populations in Ghana, India, and Nepal, the selected sites encompass diverse social, environmental, and health system contexts that are relevant to many low- and middle-income settings. Questionable in the context of India with a population of 1.46 billion, when the study is done in a small region.*

Thank you for your observation. We agree that the original wording could be interpreted as overstating the broader applicability of the findings, particularly given the substantial geographic, socioeconomic, and population diversity within and across the study countries. We have therefore revised the text to clarify that the study is conducted within selected catchment populations that were purposefully chosen to capture a broad range of demographic, socioeconomic, environmental, and health-system contexts. The revised wording emphasises that this diversity is a key strength of the study, enhancing the external validity and relevance of the findings across comparable LMIC populations and settings. We have also clarified that detailed contextual and epidemiological data, together with appropriate analytical approaches, will support assessment of the applicability of findings across different settings and inform the interpretation of study results.

Revision made in the manuscript:

“While the study is conducted within defined catchment populations in Ghana, India, and Nepal, the selected sites were chosen to capture a broad range of demographic, socioeconomic, environmental, and health-system contexts. The study aims to generate robust longitudinal evidence on NCD epidemiology, risk factors, and disease trajectories across diverse populations and settings. This diversity is a key strength of the study and enhances the external validity and generalisability of findings to populations with similar characteristics across LMIC contexts. Detailed contextual and epidemiological data, including the baseline prevalence and distribution of NCDs and associated risk factors, will support assessment of the applicability of findings across different settings. Analytical approaches such as weighting and statistical adjustment will also be applied, where supported by available population data, to account for differences between study populations and wider target populations. Findings will therefore be interpreted within their local contexts while providing insights that may inform NCD policy and programme development in comparable populations and health-system settings.” (Page 6, Lines 200-212)

17) *Lines 160-4 - The study aims to generate robust evidence on NCD epidemiology, risk factors, and disease trajectories within these populations. Detailed contextual data will enable assessment of the extent to which findings may be transferable to other settings with similar demographic, socioeconomic, and health system characteristics, thereby informing broader NCD policy and programme development. Which is dependent on the baseline prevalence of NCDs in the study area.*

We agree that the applicability of the findings to other settings will depend on the epidemiological characteristics of the study populations, including the baseline prevalence and distribution of NCDs and associated risk factors. We have revised the text, as noted in the previous comment, to clarify this point and avoid overstating the transferability of the findings.

18) *Lines Chronic 221-2 - Respiratory conditions will include self-reported history of asthma and chronic obstructive pulmonary disease (COPD), alongside assessment of lung function 22ugh peak expiratory flow measurement where feasible. – Does not make for a sound protocol with the likelihood of bias and poor comparability.*

Thank you for this comment. We acknowledge that reliance on self-reported respiratory conditions and site-specific feasibility of objective measurements may introduce measurement bias and affect comparability. However, resource and logistical constraints across study settings necessitate a pragmatic approach. We have revised the text to clarify that respiratory outcomes will be assessed using standardized protocols across sites, and that any limitations related to self-report and availability of objective measurements will be considered during analysis and interpretation, it now reads:

“Chronic respiratory conditions will include self-reported history of asthma and chronic obstructive pulmonary disease (COPD). Where feasible, lung function will also be assessed using standardised peak expiratory flow measurements conducted according to harmonised protocols across study sites. Any differences in the availability of objective measurements will be documented and considered in the interpretation of findings.” (Page 8, Lines 282-286)

19) *The protocol as written is ambitious in scope, but lacks the stated rigour-How BP would be measured, the protocol for the same, would it be a same make of a digital apparatus across sites, and modus of calibration at intervals. Same with evaluation of blood sugar, fasting or random; would it be the same make of glucometer used across settings, disposal of lancets and other material used etc.*

Thank you, it is a good idea to add more detail on measurement procedures to ensure methodological rigour and comparability across study sites. We have revised the protocol to clarify the procedures for blood pressure and blood glucose measurements, including standardised operating procedures, use of validated equipment, training of field researcher, quality assurance processes, and regular equipment calibration. We have also provided additional information on blood glucose testing procedures and biosafety measures, including the handling and disposal of lancets and other consumables.

Revision made in the manuscript:

“Blood pressure will be measured using validated automated digital blood pressure monitors following a standardised protocol across study sites, with regular equipment calibration and quality assurance procedures. Metabolic conditions will include diabetes mellitus, assessed through random blood glucose measurements and/or self-reported diagnosis, and overweight and obesity, assessed using body mass index (BMI). Blood glucose measurements will be conducted using standardised glucometers and protocols across study sites, with field researchers trained in infection prevention, biosafety procedures, and safe disposal of lancets and other sharps in accordance with local regulations.” (Page 8, Lines 274-282)

20) *How do the investigators plan on a longitudinal follow-up, which I presume would be through creation of an unique ID for each of the participants*

Participants will be assigned a unique study identification number at enrolment, which will be used to link data collected across all study waves. Contact information, linked to a unique study identification number, will be stored separately from study data and updated during follow-up visits. In addition, annual contact updates, household visits, and participant tracking procedures will be used to support longitudinal follow-up and minimise loss to follow-up. We have clarified this process in the revised manuscript:

“Each participant will be assigned a unique study identification number at enrolment. This identifier will be used to link data collected across all follow-up rounds while maintaining participant confidentiality. Participant contact information, linked to a unique study identification number, will be stored separately from study data and updated during follow-up visits to facilitate participant tracking and retention.” (Page 10-11, Lines 365-369)

21) *Data storage and security vis a vis, where would the data be housed, in which server and where, and who would have access*

We have revised the Data Management section to clarify where data will be stored and who will have access. The manuscript now specifies that data will be stored on secure encrypted servers, that identifiable information will be stored separately from study data, and that access will be restricted to authorised members of research team in accordance with institutional data governance and data protection requirements, see revised manuscript:

“Data will be stored on encrypted, password-protected servers hosted by the coordinating institution and managed in accordance with institutional data governance and data protection policies and the requirements of the UK International Data Transfer Agreement (IDTA) [24]. Participant contact information and other identifiable data (e.g., names and addresses) will be stored separately from study data under restricted access. Access to identifiable information will be limited to authorised members of the study team responsible for participant recruitment, follow-up, and data management, while de-identified datasets will be accessible only to approved investigators for analysis.” (Page 11, Lines 371-378)

22) *- There is no mention of if the necessary regulatory body clearances and approvals have been sought and permissions granted*

Thank you, this information is provided in the *Ethical Considerations* section, which states that ethical approval has been obtained from the relevant institutional review boards in Ghana, India, Nepal, and the University of Huddersfield (Reference: SREIC/2025/067). All study procedures will be conducted in accordance with relevant national and institutional requirements.

23) *In India, it would likely end up being a duplication of what the public health system has already rolled out through the NP-NCD program in which individuals > 30 years of age are screened at household and routed to care through a tiered system based on risk factor profile*

Thank you for this comment. We acknowledge that India's National Programme for Prevention and Control of Non-Communicable Diseases (NP-NCD) includes community-based screening, risk assessment, and referral pathways for adults aged 30 years and above. However, our study is designed to complement rather than duplicate these activities. In addition to screening, our study will establish a longitudinal population-based cohort of adults aged 18 years and above and collect repeated measurements to examine NCDs outcomes and risk factors, progression, multimorbidity, mental health, environmental exposures, household-level determinants, and healthcare utilisation over time. The findings will provide evidence on risk factor trajectories, vulnerable population groups, and barriers to prevention and care, which may help inform the refinement, targeting, and evaluation of NP-NCD strategies and services.

24) *There is no mention of what the next steps would be and the referral pathways if diagnosed with high blood pressure and sugars*

Thank you, see our response to Reviewer 1. As described in the Participant Safety section, participants with elevated blood pressure, raised blood glucose, or other significant findings will be informed of their results and referred to appropriate healthcare services through established local referral pathways.

25) *It also needs to be explicitly stated as to who the community workers would be who do the actual survey and measurements and whether they would be incentivized for the same*

The study will use trained research fieldworkers recruited and supervised by the local study teams and partner institutions in each country. These fieldworkers will be responsible for administering questionnaires and conducting physical measurements according to standardised protocols. They will receive comprehensive training and ongoing supervision to ensure data quality and consistency across sites. Fieldworkers will be compensated in accordance with local institutional policies and project budgets. We have clarified this in the revised manuscript:

“Data collection will be conducted by trained field researchers recruited and supervised by local study teams and partner institutions in each country. Field researchers will be responsible for administering questionnaires, obtaining informed consent, and conducting physical measurements according to standardised study protocols. All field researchers will undergo comprehensive training and regular supervision to ensure consistency and data quality across study sites. Field researchers will be remunerated in accordance with local institutional policies and project budgets.” (Page 8 Lines 264-269)

26) - *Whether the data would be shared with the public health system of the respective countries*

Thank you for this comment. De-identified aggregate findings will be disseminated to relevant public health authorities and stakeholders in each participating country to support policy and programme development. However, individual-level identifiable data will not be shared outside the study team without appropriate ethical approvals, data-sharing agreements, and compliance with national data protection regulations. We have clarified this in the manuscript:

“Study findings will be shared with participating study communities, relevant public health authorities, policymakers, and stakeholders in participating countries through reports, policy briefs, presentations, and peer-reviewed publications. De-identified and aggregated data may be shared with public health agencies and collaborating institutions in accordance with ethical approvals, data-sharing agreements, and applicable national data protection regulations.” (Page 11, Lines 378-382)

27) *- In summary the study is likely to provide more of a cross-sectional profile of risk factors rather than any definite information on the progression*

Thank you for this comment. While the baseline assessment will provide a cross-sectional profile of NCD prevalence and risk factors, the study has been designed as a longitudinal cohort with repeated follow-up assessments over four years. Specifically, comprehensive assessments will be conducted at baseline, Year 2, and Year 4, with interim follow-up visits in Years 1 and 3 to update vital status, hospitalisations, medication use, and contact information. These repeated measurements will enable the estimation of NCDs health outcomes, assessment of changes in risk factors and health behaviours over time, evaluation of multimorbidity development, and investigation of trajectories of physical and mental health. The household-based design also provides opportunities to examine how social, environmental, and household-level factors influence NCD outcomes over time.
