# Supplementary material for: A prospective longitudinal population-based cohort study of non-communicable diseases (NCDs) in Ghana, India, and Nepal (The GIN NCDs Study): A study protocol
Source: PLoS One. 2026 Nov 12;21(11):e0362114. doi: 10.1371/journal.pone.0362114 (PMC13759048; doi:10.1371/journal.pone.0362114)
Supplement: Peer review history

# Peer review: Decision Letter 0

**DOI:** 10.1371/journal.pone.0362114.r001

**Authors:** Khin Thet Wai

14 Sep 2026

Dear Dr. Lwin,

Please submit your revised manuscript by Oct 14 2026 11:59PM. If you will need more time than this to complete your revisions, please reply to this message or contact the journal office at plosone@plos.org. When you're ready to submit your revision, log on to https://www.editorialmanager.com/pone/ and select the 'Submissions Needing Revision' folder to locate your manuscript file.

- A letter that responds to each point raised by the academic editor and reviewer(s). You should upload this letter as a separate file labeled 'Response to Reviewers'.
- A marked-up copy of your manuscript that highlights changes made to the original version. You should upload this as a separate file labeled 'Revised Manuscript with Track Changes'.
- An unmarked version of your revised paper without tracked changes. You should upload this as a separate file labeled 'Manuscript'.

If you would like to make changes to your financial disclosure, please include your updated statement in your cover letter. Guidelines for resubmitting your figure files are available below the reviewer comments at the end of this letter.

If applicable, we recommend that you deposit your laboratory protocols in protocols.io to enhance the reproducibility of your results. Protocols.io assigns your protocol its own identifier (DOI) so that it can be cited independently in the future. For instructions see: https://journals.plos.org/plosone/s/submission-guidelines#loc-laboratory-protocols. Additionally, PLOS One offers an option for publishing peer-reviewed Lab Protocol articles, which describe protocols hosted on protocols.io. Read more information on sharing protocols at https://plos.org/protocols?utm_medium=editorial-email&utm_source=authorletters&utm_campaign=protocols.

As the corresponding author, your ORCID iD is verified in the submission system and will appear in the published article. PLOS supports the use of ORCID, and we encourage all coauthors to register for an ORCID iD and use it as well. Please encourage your coauthors to verify their ORCID iD within the submission system before final acceptance, as unverified ORCID iDs will not appear in the published article. *Only*  the individual author can complete the verification step; PLOS staff *cannot*  verify ORCID iDs on behalf of authors.

We look forward to receiving your revised manuscript.

Kind regards,

Khin Thet Wai, MBBS, MPH, MA

Academic Editor

PLOS One

Journal Requirements:

When submitting your revision, we need you to address these additional requirements.

1. Please ensure that your manuscript meets PLOS ONE's style requirements, including those for file naming. The PLOS ONE style templates can be found at

https://journals.plos.org/plosone/s/file?id=wjVg/PLOSOne_formatting_sample_main_body.pdf and

https://journals.plos.org/plosone/s/file?id=ba62/PLOSOne_formatting_sample_title_authors_affiliations.pdf

2. Please include a complete copy of PLOS’ questionnaire on inclusivity in global research in your revised manuscript. Our policy for research in this area aims to improve transparency in the reporting of research performed outside of researchers’ own country or community. The policy applies to researchers who have travelled to a different country to conduct research, research with Indigenous populations or their lands, and research on cultural artefacts. The questionnaire can also be requested at the journal’s discretion for any other submissions, even if these conditions are not met. Please find more information on the policy and a link to download a blank copy of the questionnaire here: https://journals.plos.org/plosone/s/best-practices-in-research-reporting. Please upload a completed version of your questionnaire as Supporting Information when you resubmit your manuscript.

3. Thank you for stating the following financial disclosure: [This study is funded by the university research fund from the University of Huddersfield].

Please state what role the funders took in the study.  If the funders had no role, please state: ""The funders had no role in study design, data collection and analysis, decision to publish, or preparation of the manuscript.""

If this statement is not correct you must amend it as needed.

Please include this amended Role of Funder statement in your cover letter; we will change the online submission form on your behalf.

4. When completing the data availability statement of the submission form, you indicated that you will make your data available on acceptance. We strongly recommend all authors decide on a data sharing plan before acceptance, as the process can be lengthy and hold up publication timelines. Please note that, though access restrictions are acceptable now, your entire data will need to be made freely accessible if your manuscript is accepted for publication. This policy applies to all data except where public deposition would breach compliance with the protocol approved by your research ethics board. If you are unable to adhere to our open data policy, please kindly revise your statement to explain your reasoning and we will seek the editor's input on an exemption. Please be assured that, once you have provided your new statement, the assessment of your exemption will not hold up the peer review process.

5. Please amend either the title on the online submission form (via Edit Submission) or the title in the manuscript so that they are identical.

6. Please upload a copy of Supporting Information Figure/Table/etc. [S1 Fig. Directed acyclic graph of risk factors for non-communicable diseases (NCDs) outcomes.] which you refer to in your text on page 12.

7. Please include captions for your Supporting Information files at the end of your manuscript, and update any in-text citations to match accordingly. Please see our Supporting Information guidelines for more information: http://journals.plos.org/plosone/s/supporting-information.

8. If the reviewer comments include a recommendation to cite specific previously published works, please review and evaluate these publications to determine whether they are relevant and should be cited. There is no requirement to cite these works unless the editor has indicated otherwise.

Additional Editor Comments:

This manuscript touches an interesting topic. However, the authors should improve scientific integrity of this study protocol by doing an extensive revision concerning justification, methododology, and discussion points.

[Note: HTML markup is below. Please do not edit.]

Reviewers' comments:

Reviewer's Responses to Questions

**Comments to the Author**

1. Does the manuscript provide a valid rationale for the proposed study, with clearly identified and justified research questions?

Reviewer #1: Partly

Reviewer #2: Partly

**********

2. Is the protocol technically sound and planned in a manner that will lead to a meaningful outcome and allow testing the stated hypotheses?

Reviewer #1: Yes

Reviewer #2: Partly

**********

3. Is the methodology feasible and described in sufficient detail to allow the work to be replicable?

Reviewer #1: Yes

Reviewer #2: No

**********

4. Have the authors described where all data underlying the findings will be made available when the study is complete?

The PLOS Data policy

Reviewer #1: Yes

Reviewer #2: Yes

**********

5. Is the manuscript presented in an intelligible fashion and written in standard English?

Reviewer #1: Yes

Reviewer #2: Yes

**********

Please use the space provided to explain your answers to the questions above and, if applicable, provide comments about issues authors must address before this protocol can be accepted for publication. You may also include additional comments for the author, including concerns about research or publication ethics.

You may also provide optional suggestions and comments to authors that they might find helpful in planning their study.

Reviewer #1: Introduction:

- Why did you choose Ghana, India and Nepal out of all LMICs?

- What are similarities and differences in risk factors and NCDs among them? Will you be comparing the results across these countries? Why do you think they should be compared?

Methodology:

- When reading about India, I think of two big topics related to NCDs are air pollution and rich-poor gap among populations. Do other two countries have similar backgrounds?

o I believe air pollution is an exposure in your study with risk factors of secondhand smoke and solid fuel. Did you also take account of outdoor air pollution?

o Line 132 and 133: These sites have been chosen to reflect a range of geographic, socioeconomic, and health system contexts, enabling the study to capture variation in exposure to risk factors and access to care. I would love to know more about socioeconomic considerations and which areas were selected in each country to account for it.

- What age‑group categories are you planning to use in the study?

- Will individuals with existing diseases be included?

- How do you make sure that you are capturing related risk factors for each disease? For example, does your mental health outcome fully capture the spectrum of mental health disorders when assessed only through the WHO STEPS depression and suicide modules? Do you have subject matter experts for specific diseases who can advise?

- Do you also take account of intersectional considerations between risk factors and NCDs?

- Will there be a benefit or incentive for participants? Could this affect the study in any way?

- What procedures are in place for participants who are found to have elevated blood pressure or raised blood glucose during a measurement but are not currently receiving treatment? Additionally, how will cases of suicidal ideation identified through the survey be managed?

Discussion:

- What age and sex related recruitment biases do you anticipate, and how might these influence the study findings?

- Are there any other limitations and potential bias that should be taken into consideration in interpreting results of your study? Could there be social desirability bias due to participants self‑reporting their risk factors?

- It was said that a key strength of the study is its household based design, which allows for the examination of shared exposures and family level influences. This appears to be a distinctive and potentially novel aspect. It would be helpful to expand on this component by providing more background from the literature on household level determinants of NCDs and explaining how you plan to analyze these relationships. Additionally, outlining the specific hypotheses related to household clustering, shared environmental or behavioral exposures, and intra family variation in risk factors or outcomes would strengthen this section.

Reviewer #2: I read with interest the manuscript titled 'A population-based cohort study of non-communicable diseases (NCDs) in Ghana, India, and Nepal (The GIN NCDs Study): A study protocol'

Line 45 - This study will generate longitudinal evidence on NCD risk factors, multimorbidity, and mental health in LMICs, addressing critical data gaps.

Lines 116-117 At Year 0 (Baseline), a comprehensive assessment will be conducted, including demographic, 116 behavioural, clinical, and anthropometric measurements. Need to specify what the clinical parameters would be.

Lines 158-9 -While the study is conducted within defined catchment populations in Ghana, India, and Nepal, the selected sites encompass diverse social, environmental, and health system contexts that are relevant to many low- and middle-income settings. Questionable in the context of India with a population of 1.46 billion, when the study is done in a small region.

Lines 160-4 - The study aims to generate robust evidence on NCD epidemiology, risk factors, and disease trajectories within these populations. Detailed contextual data will enable assessment of the extent to which findings may be transferable to other settings with similar demographic, socioeconomic, and health system characteristics, thereby informing broader NCD policy and programme development. Which is dependent on the baseline prevalence of NCDs in the study area.

Lines Chronic 221-2 - Respiratory conditions will include self-reported history of asthma and chronic obstructive pulmonary disease (COPD), alongside assessment of lung function 22ugh peak expiratory flow measurement where feasible. – Does not make for a sound protocol with the likelihood of bias and poor comparability.

The protocol as written is ambitious in scope, but lacks the stated rigour-

- How BP would be measured, the protocol for the same, would it be a same make of a digital apparatus across sites, and modus of calibration at intervals

- Same with evaluation of blood sugar, fasting or random; would it be the same make of glucometer used across settings, disposal of lancets and other material used etc

- How do the investigators plan on a longitudinal follow-up, which I presume would be through creation of an unique ID for each of the participants

- Data storage and security vis a vis, where would the data be housed, in which server and where, and who would have access

- There is no mention of if the necessary regulatory body clearances and approvals have been sought and permissions granted

- In the context of India, it would likely end up being a duplication of what the public health system has already rolled out through the NP-NCD program in which individuals > 30 years of age are screened at household and routed to care through a tiered system based on risk factor profile

- There is no mention of what the next steps would be and the referral pathways if diagnosed with high blood pressure and sugars

- It also needs to be explicitly stated as to who the community workers would be who do the actual survey and measurements and whether they would be incentivized for the same

- Whether the data would be shared with the public health system of the respective countries

- In summary the study is likely to provide more of a cross-sectional profile of risk factors rather than any definite information on the progression

**********

what does this mean?). If published, this will include your full peer review and any attached files.

If you choose “no”, your identity will remain anonymous but your review may still be made public.

**Do you want your identity to be public for this peer review?**  For information about this choice, including consent withdrawal, please see our Privacy Policy

Reviewer #1: No

Reviewer #2: **Yes:**  Jaideep C Menon

**********

[NOTE: If reviewer comments were submitted as an attachment file, they will be attached to this email and accessible via the submission site. Please log into your account, locate the manuscript record, and check for the action link "View Attachments". If this link does not appear, there are no attachment files.]

To ensure your figures meet our technical requirements, please review our figure guidelines: https://journals.plos.org/plosone/s/figures

You may also use PLOS’s free figure tool, NAAS, to help you prepare publication quality figures: https://journals.plos.org/plosone/s/figures#loc-tools-for-figure-preparation.

NAAS will assess whether your figures meet our technical requirements by comparing each figure against our figure specifications.

# Author comment: Author response to Decision Letter 1

**DOI:** 10.1371/journal.pone.0362114.r002

13 Oct 2026

Dear Editorial Team,

I am writing to submit a revision of the above manuscript. We sincerely appreciate the helpful comments from the reviewer, and we have revised the manuscript according to his/her suggestions.

The modified sections in the manuscript are highlighted using tracked changes. Please find the attached response to the reviewer which details the changes we have made along with explanations for these changes.

We hope that the revised manuscript will now be acceptable for publication in PLOS One and look forward to your decision. Thank you very much for your consideration of this manuscript.

Yours sincerely,

Dr Kaung Suu Lwin, MBBS MHSc PhD

School of Human and Health Sciences

University of Huddersfield | Queensgate | Huddersfield | HD1 3DH

Attachment Submitted filename: Response letter_LCS protocol 13102026.docx

# Peer review: Decision Letter 1

**DOI:** 10.1371/journal.pone.0362114.r003

**Authors:** Khin Thet Wai

20 Oct 2026

A prospective longitudinal population-based cohort study of non-communicable diseases (NCDs) in Ghana, India, and Nepal (The GIN NCDs Study): A study protocol

PONE-D-26-31442R1

Dear Dr. Lwin,

We’re pleased to inform you that your manuscript has been judged scientifically suitable for publication and will be formally accepted for publication once it meets all outstanding technical requirements.

Within one week, you’ll receive an e-mail detailing the required amendments. When these have been addressed, you’ll receive a formal acceptance letter and your manuscript will be scheduled for publication.

An invoice will be generated when your article is formally accepted. Please note, if your institution has a publishing partnership with PLOS and your article meets the relevant criteria, all or part of your publication costs will be covered. Please make sure your user information is up-to-date by logging into Editorial Manager at Editorial Manager® and clicking the ‘Update My Information' link at the top of the page. For questions related to billing, please contact billing support.

If your institution or institutions have a press office, please notify them about your upcoming paper to help maximize its impact. If they’ll be preparing press materials, please inform our press team as soon as possible -- no later than 48 hours after receiving the formal acceptance. Your manuscript will remain under strict press embargo until 2 pm Eastern Time on the date of publication. For more information, please contact onepress@plos.org.

Kind regards,

Khin Thet Wai, MBBS, MPH, MA

Academic Editor

PLOS One

Additional Editor Comments (optional):

All comments are adequately addressed.

Reviewers' comments:

Reviewer's Responses to Questions

**Comments to the Author**

1. Does the manuscript provide a valid rationale for the proposed study, with clearly identified and justified research questions?

Reviewer #2: Yes

**********

2. Is the protocol technically sound and planned in a manner that will lead to a meaningful outcome and allow testing the stated hypotheses?

Reviewer #2: Yes

**********

3. Is the methodology feasible and described in sufficient detail to allow the work to be replicable?

Reviewer #2: Yes

**********

4. Have the authors described where all data underlying the findings will be made available when the study is complete?

The PLOS Data policy

Reviewer #2: Yes

**********

5. Is the manuscript presented in an intelligible fashion and written in standard English?

Reviewer #2: Yes

**********

Please use the space provided to explain your answers to the questions above and, if applicable, provide comments about issues authors must address before this protocol can be accepted for publication. You may also include additional comments for the author, including concerns about research or publication ethics.

You may also provide optional suggestions and comments to authors that they might find helpful in planning their study.

Reviewer #2: All the concerns raised by the reviewers have been suitably addressed, I wish the authors the best with their research

**********

what does this mean?). If published, this will include your full peer review and any attached files.

If you choose “no”, your identity will remain anonymous but your review may still be made public.

**Do you want your identity to be public for this peer review?**  For information about this choice, including consent withdrawal, please see our Privacy Policy

Reviewer #2: **Yes:**  Jaideep C Menon

**********

# Editor report: Acceptance letter

**DOI:** 10.1371/journal.pone.0362114.r004

**Authors:** Khin Thet Wai

PONE-D-26-31442R1

PLOS One

Dear Dr. Lwin,

I'm pleased to inform you that your manuscript has been deemed suitable for publication in PLOS One. Congratulations! Your manuscript is now being handed over to our production team.

At this stage, our production department will prepare your paper for publication. This includes ensuring the following:

* All references, tables, and figures are properly cited

* All relevant supporting information is included in the manuscript submission,

* There are no issues that prevent the paper from being properly typeset

You will receive further instructions from the production team, including instructions on how to review your proof when it is ready. Please keep in mind that we are working through a large volume of accepted articles, so please give us a few days to review your paper and let you know the next and final steps.

Lastly, if your institution or institutions have a press office, please let them know about your upcoming paper now to help maximize its impact. If they'll be preparing press materials, please inform our press team within the next 48 hours. Your manuscript will remain under strict press embargo until 2 pm Eastern Time on the date of publication. For more information, please contact onepress@plos.org.

You will receive an invoice from PLOS for your publication fee after your manuscript has reached the completed accept phase. If you receive an email requesting payment before acceptance or for any other service, this may be a phishing scheme. Learn how to identify phishing emails and protect your accounts at https://explore.plos.org/phishing.

If we can help with anything else, please email us at customercare@plos.org.

Thank you for submitting your work to PLOS One and supporting open access.

Kind regards,

PLOS One Editorial Office Staff

on behalf of

Dr. Khin Thet Wai

Academic Editor

PLOS One
